# Genomic characterisation of Crimean-Congo haemorrhagic fever virus (CCHFV) in Tajikistan identifies a novel reassortant virus

**DOI:** 10.1371/journal.pntd.0014204

**Published:** 2026-04-07

**Authors:** Jake D’Addiego, Farida Tishkova, Manija Mullojonova, Viktoriya Dzhuraeva, Grant Lythe, Stuart Dent, Carmen Molina-París, Thomas Leitner, Roger Hewson

**Affiliations:** 1 Department of Infection Biology, Faculty of Infectious and Tropical Diseases, London School of Hygiene and Tropical Medicine, London, United Kingdom; 2 Public Health Microbiology Directorate, UK Health Security Agency, Salisbury, United Kingdom; 3 Virology Laboratory, Tajik Research Institute of Preventative Medicine, Dushanbe, Tajikistan; 4 Department of Applied Mathematics, University of Leeds, Leeds, United Kingdom; 5 Theoretical Biology and Biophysics Group, Theoretical Division, Los Alamos National Laboratory, Los Alamos, New Mexico, United States of America; 6 Genomic Surveillance Unit, Wellcome Sanger Institute, Hinxton, United Kingdom; Oregon State University College of Veterinary Medicine, UNITED STATES OF AMERICA

## Abstract

Crimean-Congo haemorrhagic fever virus (CCHFV) is an important human tick-borne pathogen, able to cause severe haemorrhagic fever. CCHFV is endemic in Tajikistan, which records between 5–38 cases of CCHF a year from southern regions. Molecular surveillance of CCHFV is crucial to implement effective prevention and control strategies, understand viral evolution, study transmission dynamics, and develop effective diagnostics, therapeutics, and vaccines. While the presence of Asia-1 and Asia-2 genotypes has been previously reported, only two historical samples from Tajikistan have been fully sequenced. In this study we developed and applied a genotype IV-specific tiling PCR enrichment approach recovering 52 CCHFV genome segment sequences from clinical and *Hyalomma* tick samples collected between 2017–2023. Most sequences belonged to the Asia-2 genotype, but one virus exhibited an Asia-1 S segment combined with Asia-2 M and L segments, representing the first evidence of such viral reassortment event in Tajikistan.

## Author summary

Crimean-Congo haemorrhagic fever (CCHF) is a severe haemorrhagic disease caused by CCHF virus. The virus is found in Europe, Africa and Asia, and is predominantly transmitted by *Hyalomma* ticks. The negative-sense RNA genome is highly diverse and consists of the small (S), medium (M) and large (L) segments. Traditionally, different CCHFV genotypes have been named after their main geographic location, with Central Asian countries mostly reporting the presence of Asia-1 and Asia-2 viruses (genotypes IVa and IVb). In this study, we have developed a novel PCR-based enrichment methodology designed to amplify genotype IV CCHFV genome segments and characterised viruses from clinical as well as tick samples collected from Tajikistan, an endemic region for the virus with limited genomic output. Furthermore, we described for the first time in the region a novel clinical reassortant CCHFV containing an Asia-1 S segment and Asia-2 M and L segments, further elucidating previously unknown genetic diversity within Tajikistan. The genetic characterisation of circulating viruses from endemic regions is crucial to develop and implement local public health strategies aimed at preventing and controlling virus transmission, as well as develop effective diagnostics, therapeutics and vaccines.

## Introduction

Crimean-Congo haemorrhagic fever virus (CCHFV) is a zoonotic tick-borne human pathogen belonging to the *Nairoviridae* family (order *Hareavirales*) [1]. The virus is endemic in Europe, Africa, and Asia, and closely follows the geographic distribution of its main vector and reservoir, ticks from the genus *Hyalomma*. Experimental studies have shown that ticks from the genus *Rhipicephalus* are also competent CCHFV vectors [2] and the main drivers of CCHF transmission in regions where *Hyalomma* ticks are uncommon [3,4]. CCHFV is usually transmitted to humans through the bite of infected ticks, although transmission through direct exposure to viraemic bodily fluids from patients or animals has also been reported [3]. The virus contains a tripartite, negative-sense RNA genome consisting of the small (S), medium (M), and large (L) segments encoding for the nucleocapsid protein (NP), the precursor to the viral glycoproteins (GPC), and the RNA-dependent RNA polymerase (RDRP), respectively [5]. Traditionally, based on phylogenetic analysis of the S segment, there are seven different CCHFV genotypes, however due to genomic segment reassortment, which has been widely described in the literature [5–8], this number differs for other genome segments [9].

While most CCHF infections are asymptomatic or cause mild non-specific symptoms [10], a proportion progress to severe and often fatal haemorrhagic fever. Due to the lack of licensed vaccines or therapeutics for CCHF, the World Health Organisation (WHO) has previously listed CCHFV as a priority pathogen for accelerated research [11]. The recent reports of CCHF outbreaks in Europe, Africa, and Asia [12–18], and risk alerts issued in Spain and Greece [19], highlight the necessity for continued CCHFV surveillance in endemic regions. A revision of the WHO 2019 CCHF R&D roadmap published in 2024 has laid out key research priorities, including genetic characterisation and molecular surveillance of the virus in humans, animals, and vectors [20].

Most CCHFV cases in Asia are reported from central Asian countries including Kazakhstan, Kyrgyzstan, Tajikistan, Turkmenistan, and Uzbekistan [21] where CCHFV is endemic. The detection of both Asia-1 and Asia-2 (genotype IVa and IVb) CCHF viruses has been previously reported in Kazakhstan [22,23], Uzbekistan (Asia-1) [24], and Turkmenistan (Asia-2) [9].

In Tajikistan, the national system for epidemiological surveillance of infectious diseases is coordinated by the Ministry of Health and Social Protection of the Population through the country’s sanitary-epidemiological surveillance network. CCHF is among the notifiable diseases subject to mandatory epidemiological monitoring. Since 1961, CCHFV surveillance and formal diagnosis in Tajikistan have been undertaken mainly by the Ministry of Health’s Tajik Research Institute of Preventative Medicine (TRIPM), in Dushanbe [25]. The majority of CCHFV infections in Tajikistan occur in the southern region, likely due to favourable environmental factors for vector establishment [26] often following bites from *H. anatolicum* ticks [25]. The country currently records between 5–38 CCHF cases per year [21]. Previous studies aimed at genetically characterising CCHFV in Tajikistan have found evidence of both Asia-1 and Asia-2 viruses circulating in the region based on S segment sequence data analysis [26,27].

Recently, we have developed a tiling, multiplex PCR-based approach for recovery of Europe 1 (genotype V) CCHFV genomes which has shown better genome recovery from serum samples with low virus titre (< 10^6^ copies/mL) over non-targeted enrichment strategies [28]. The method was successfully implemented in a public health laboratory to genetically characterise viruses associated with recent clinical cases of CCHF [29].

In this study we have developed and applied a novel amplicon-based tiling scheme specific for Asian CCHFV genotypes, and genetically characterised viruses from clinical samples and *H. anatolicum* ticks collected in Tajikistan between 2017 and 2023. While most of the sequences generated in this study clustered within the Asia-2 genotype, we report the detection of an S segment Asia-1 reassortant virus from a clinical sample which has not been previously reported from the region. Prior to this study, only 28 published CCHFV genomic segment sequences from the region were available on the Bacterial and Viral Bioinformatics Resource Center [30] (accessed in September 2025), with no records having been published in the last decade. Our efforts, which aimed at genetically characterising CCHF viruses in Tajikistan from both clinical as well as tick samples extend our understanding of CCHFV epidemiology and transmission dynamics within the region. Furthermore, our data contributes to the broader global understanding of CCHFV, aiding in the development of effective public health strategies to predict and prevent future outbreaks which continue to pose threats to human health.

## Methods

### Ethics statement

The study was approved by the local Bioethical Council at the National Academy of Sciences of Tajikistan (proposal 2109432). All study participants provided an informed written consent form.

### Sample collection, RNA extraction and quantification

**Fig 1:**
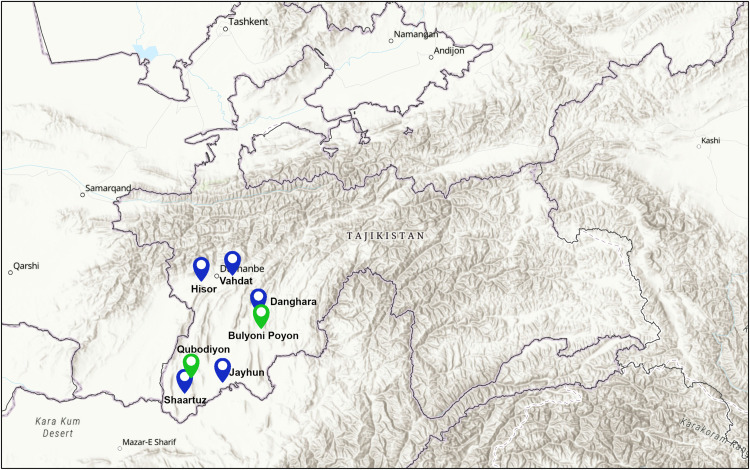
Sampling locations for the clinical (blue pins) and tick-derived (green pins) samples. Map image was generated using ArcGIS software [31,32,33].

All samples were collected by TRIPM as part of Tajikistan’s national system for epidemiological surveillance of CCHFV. Clinical serum samples were collected between 23 June 2017 and 12 July 2023 and inactivated in AVL buffer (QIAGEN) as previously described [28]. Tick samples were collected by flagging between 7 May 2023 and 26 May 2023, washed in 300 µL of PBS and homogenised using a mortar and pestle. Tick collections were conducted primarily in Bulyoni Poyon and nearby areas where recent human cases had been reported and where *H. anatolicum* populations are abundant. All samples were processed by TRIPM Virology Laboratory following national CCHFV guidelines and legislation. Sampling locations, which represent the principal endemic focus rather than a uniform national sampling distribution, have been indicated in Fig 1.

RNA was extracted with the QIAamp Viral RNA kit (QIAGEN) following the manufacturer’s spin protocol and eluted in 60 µL of AVE buffer. RNA was shipped to the UK Health Security Agency (UKHSA) and stored at -80 °C. RT-qPCR was performed on duplicate 10-fold dilutions of the RNA extracts as previously described [34]. Copies of CCHFV genomic RNA were determined based on a quantified synthetic S segment RNA standard curve and normalised taking into account extraction and elution volumes. 11 tick homogenate samples and 11 clinical serum samples with a cycle threshold (CT) value between 23.7 and 34.3 were included in the study (Table 1). These sequenced samples represent a subset of RT-PCR–positive detections identified through the national surveillance programme during the study period; comprehensive surveillance statistics will be reported separately.

**Table 1:** CT values and RNA copy numbers of CCHFV clinical and tick samples. *Samples collected from patients with reported contact to C18.

Sample ID	Isolate Host				Sample ID	Isolate Host			
		City	CT	RNA copies (mL or tick)			City	CT	RNA copies (mL or tick)
C7	Human	Hisor	29.3	8.91 x 10^5^	6_161	*H. anatolicum*	Bulyoni Poyon	30.2	7.63 x 10^4^
C14	Human	Vahdat	29.8	6.39 x 10^5^	14_169	*H. anatolicum*	Bulyoni Poyon	27.8	3.77 x 10^5^
C21*	Human	Jayhun	27.9	2.36 x 10^6^	4_159	*H. anatolicum*	Bulyoni Poyon	30.9	4.72 x 10^4^
C16	Human	Jayhun	26.8	5.03 x 10^6^	12_167	*H. anatolicum*	Bulyoni Poyon	32.4	1.71 x 10^4^
C17	Human	Shaartuz	23.7	3.90 x 10^7^	23_178	*H. anatolicum*	Bulyoni Poyon	33.3	9.31 x 10^3^
C6	Human	Danghara	27.0	4.35 x 10^6^	24_24	*H. anatolicum*	Qubodiyon	34.0	1.15 x 10^4^
C12	Human	Shaartuz	33.9	3.96 x 10^4^	26_26	*H. anatolicum*	Qubodiyon	34.3	9.28 x 10^3^
C18	Human	Jayhun	34.0	4.01 x 10^4^	27_27	*H. anatolicum*	Qubodiyon	34.3	9.61 x 10^3^
C19*	Human	Jayhun	33.9	4.11 x 10^4^	22_177	*H. anatolicum*	Bulyoni Poyon	33.4	9.04 x 10^3^
C20*	Human	Jayhun	33.6	4.90 x 10^4^	24_179	*H. anatolicum*	Bulyoni Poyon	33.7	6.57 x 10^3^
C25	Human	Danghara	33.5	6.11 x 10^4^	30_185	*H. anatolicum*	Bulyoni Poyon	33.4	9.11 x 10^3^

RNA copy numbers were estimated from the determined CT values. Clinical samples contained between 3.96 x 10^4^ and 3.90 x 10^7^ RNA copies/mL of sample. The tick homogenate samples contained between 6.57 x 10^3^ and 3.77 x 10^5^ RNA copies/tick.

Accession numbers and reference coverages of the generated 52 genomic segment sequences are presented in Table 2.

### Targeted enrichment

Considering the low viraemia of the processed samples (average CT values of 30.31 and 32.52 for the clinical and tick samples respectively) a targeted, amplicon-based sequencing approach was utilised as previously described [28] due to its higher performance for genome recovery in low virus titre material. Copy DNA was synthesised from either 1:10 (serum samples) or 1:2 (tick homogenates) dilutions of the RNA extracts. Two primer pools for CCHFV S and L genome segments, and four primer pools for the M segment were prepared, with each pool containing primers targeting alternate 500 bp fragments designed using the Primal Scheme software [35]. Details of each primer pool with final primers concentrations are provided in the S1 and S2 Tables.

### Non-targeted enrichment

A single high-titre clinical sample (C17) was processed with sequence-independent single-primer amplification (SISPA) to provide a high-fidelity reference genome for targeted enrichment methodology optimisation. The sample was selected as its CT value of 23.7 was within our previously established sensitivity range for near complete genome recovery using the methodology [28]. 44 µL of a 2.7-fold dilution of the RNA extract was treated with TURBO DNase (Thermo Fisher Scientific) prior to SISPA amplification as previously described [28].

### Sequencing library preparation

Illumina (sample C17) and nanopore (all other samples) sequencing libraries were prepared as previously described [28]. Illumina sequencing was carried out on a MiSeq instrument operated by the UKHSA Colindale Sequencing Laboratory.

### Bioinformatic analysis

Raw Illumina sequencing reads were processed as previously described [28]. Illumina reads were mapped against reference sequences (accession number KJ676542.1 for the S segment, ON500501.1 or KC344856.1 for the M segment, and KX013456.1 for the L segment) utilising BWA MEM [36]. Nanopore sequencing data was filtered based on quality (minimum 8). Nanopore sequencing reads were mapped against the reference utilising minimap2 [37].

Consensus sequences were derived from mapped reads using BCFtools [38] masking regions with no coverage. The dual primer pool barcoding used for sequencing each sample allowed for consensus sequences to be called only from regions which displayed the expected tiling amplification pattern, excluding regions affected by background contamination. For targeted enrichment data, primer sequences were first removed from sorted BAM files with BAMClipper [39] prior to consensus generation. Reported consensus reference coverages (Table 2) were calculated excluding gaps.

**Table 2:** Reference coverage and genotype of the analysed CCHFV samples. *Sample RNA enriched with SISPA followed by Illumina sequencing.

Sample ID	S Segment			M segment			L segment		
	Accession Number	Reference Coverage	Genotype	Accession Number	Reference Coverage	Genotype	Accession Number	Reference Coverage	Genotype
C7	PV390604	100.00	IVb/Asia 2	PV390615	99.34	IVb/Asia 2	PV390622	99.99	IVb/Asia 2
C14	PV390605	100.00	IVa/Asia 1	PV390616	99.49	IVb/Asia 2	PV390623	97.68	IVb/Asia 2
C21	PV390608	100.00	IVb/Asia 2	PV390619	92.44	IVb/Asia 2	PV390626	97.42	IVb/Asia 2
C16	PV390606	100.00	IVb/Asia 2	PV390617	99.66	IVb/Asia 2	PV390624	100.00	IVb/Asia 2
C17*	PV390607	100.00	IVb/Asia 2	PV390618	99.55	IVb/Asia 2	PV390625	100.00	IVb/Asia 2
C6	PV390603	100.00	IVb/Asia 2	PV390614	97.42	IVb/Asia 2	PV390621	100.00	IVb/Asia 2
C12	PV953843	74.66	IVb/Asia 2	PV953844	83.24	IVb/Asia 2	PV953845	99.93	IVb/Asia 2
C18	PV953846	100.00	IVb/Asia 2	PV953847	91.80	IVb/Asia 2	PV953848	99.93	IVb/Asia 2
C19	PV953849	100.00	IVb/Asia 2	PV953850	91.82	IVb/Asia 2	PV953851	99.96	IVb/Asia 2
C20	PV953852	99.52	IVb/Asia 2	PV953853	83.61	IVb/Asia 2	PV953854	99.97	IVb/Asia 2
C25	PV953855	100.00	IVb/Asia 2				PV953856	99.96	IVb/Asia 2
6_161	PV390610	100.00	IVb/Asia 2				PV390627	100.00	IVb/Asia 2
14_169	PV390612	74.96	IVb/Asia 2						
4_159	PV390609	54.93	IVb/Asia 2						
12_167	PV390611	74.60	IVb/Asia 2						
23_178	PV390613	54.87	IVb/Asia 2	PV390620	37.93	IVb/Asia 2			
24_24	PV738566	55.35	IVb/Asia 2				PV738567	64.26	IVb/Asia 2
26_26	PV738568	74.00	IVb/Asia 2				PV738569	75.48	IVb/Asia 2
27_27	PV738570	74.12	IVb/Asia 2				PV738571	79.50	IVb/Asia 2
22_177	PV738572	72.68	IVb/Asia 2	PV738573	91.26	IVb/Asia 2	PV738574	70.61	IVb/Asia 2
24_179	PV738575	29.41	IVb/Asia 2						
30_185	PV953857	99.88	IVb/Asia 2	PV953858	90.04	IVb/Asia 2	PV953859	97.45	IVb/Asia 2

12 S segment, 5 M segment, and 13 L segment sequences contained complete coding regions. Partial coding sequences were retained for genotypes assignment and phylogenetic analysis where they met the predefined completeness threshold described above. Overall, 9 samples yielded near-complete genomes (≥ 90% reference coverage across all three segments). A single S segment sequence (PV390605) clustered within the Asia-1 genotype (Table 2).

All generated consensus sequences have been deposited in GenBank with the following accession numbers: PV390603- PV390627; PV738566- PV738575; PV953843-PV953859.

### Genotype characterisation and pairwise identity determination

Maximum likelihood phylogenies were generated with MEGA 11 [40] applying a general time reversible model. Bootstrap support values were generated with 1000 replicates. Sequences were aligned using MUSCLE [41] and trimmed to exclude low coverage regions resulting in final alignment lengths of 919 bp (S segment), 4467 bp (M segment), and 11831 bp (L segment) including gaps. Phylogenetic analysis included sequences with at least 70% (S segment) or 90% (M and L segments) reference coverage. A lower reference coverage threshold for the S segment was utilised to allow phylogenetic analysis of informative tick-derived sequences. Genotypes of partial sequences were determined based on nucleotide similarity to other published sequences using Basic Local Alignment Search Tool (BLAST) [42]. Pairwise identities (%) were determined with Geneious Prime software (version 2023.0.2).

## Results

Genomic segment recovery from both Asia-1 and Asia-2 lineages was achieved for clinical as well as tick homogenate samples with the newly developed tiling multiplex PCR methodology.

### Phylogenetic analysis of the S, M and L segments confirms detection of an S segment Asia-1 reassortant virus in a clinical sample

**Fig 2:**
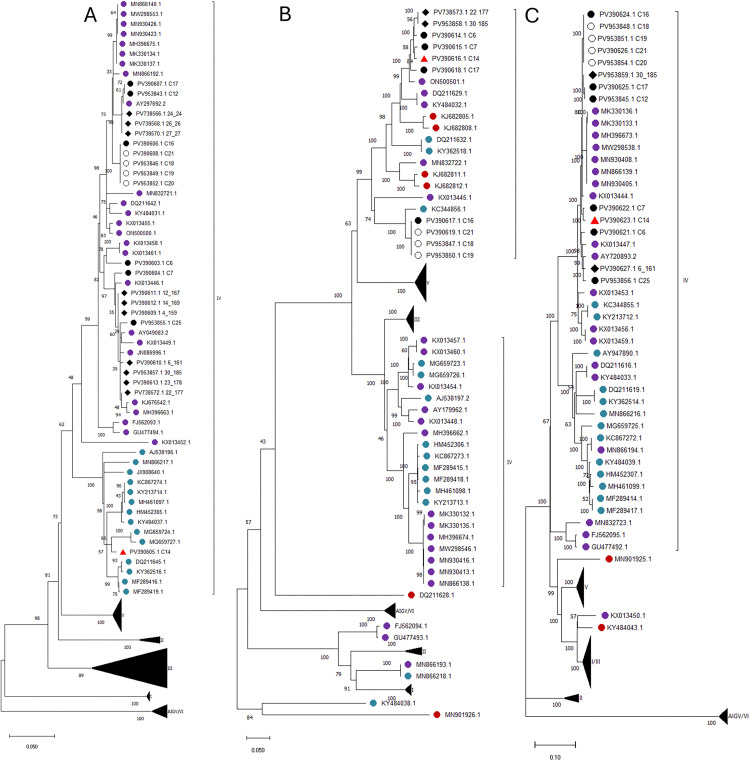
Maximum likelihood phylogeny for the S (A), M (B) and L (C) genomic segments. Sequences have been colour-coded based on genotype affiliation of the S segment as follows: III (Africa-3, red), IV (Asia-1/IVa, blue; Asia-2/IVb, purple). Sequences generated in this study are indicated by black/white circles (clinical samples) or black rhombi (tick homogenate samples). White circles indicate sequences recovered from patients with reported contact. Sequences belonging to the identified Asia-1 S segment reassortant virus are indicated by red triangles. Genotype numbers are indicated next to the relevant clades. Except for single nodes which have been colour coded as described above, clades not relevant to this study have been compressed (black triangles).

Except for a single clinical S segment sequence (PV390605) derived from sample C14 which clustered with an Asia-1 (genotype IVa) 2017 strain from China (MG659724.1), all other generated S segment sequences clustered within the Asia-2 (genotype IVb) clade, supported by high bootstrap values (Fig 2A).

Notably, the M (PV390616.1) and L (PV390623.1) segment sequences derived from sample C14 both clustered within the Asia-2 genotype (Fig 2B and 2C), indicating reassortment of the Asia-1 S segment.

Inspection of mapped reads revealed no mixed nucleotide positions above the 5% minor-allele frequency in the reassortant sample (C14). Read-depth profiles were even across all three segments and no dual mapping signatures were observed. These findings indicate the presence of single, homogeneous viral populations, supporting reassortment rather than dual infection as the most plausible explanation.

The S segment sequences derived from two clinical samples and three tick samples clustered with a sequence derived from a clinical sample isolated in Tajikistan in 1990 (AY297692.2). Five clinical samples all isolated in Jayhun formed a separate cluster, with the closest sequence also being AY297692.2. A clinical sample (PV390603.1) was more closely related to a 1973 tick S segment sequence from Turkmenistan. All other S segment sequences clustered with previously published sequences derived from clinical samples isolated in Central and South Asia (Fig 2A). A single partial Asia-2 S segment sequence (PV738575.1) was excluded from phylogenetic analysis due to low reference coverage.

All M segment sequences generated in this study cluster within the Asia-2 genotype with high bootstrap support (Fig 2B). M segment sequences derived from the tick samples as well as four clinical samples cluster with a 2021 tick sequence from China (ON500501.1), whilst M segment sequences from the other four clinical samples cluster with a 2012 clinical sample sequence from Afghanistan (KC344856.1). Three partial Asia-2 M segment sequences (PV953844, PV953853, PV390620) were excluded from phylogenetic analysis due to low reference coverage.

All L segment sequences generated in this study also cluster with other Asia-2 genotype (Fig 2C). Most L segment sequences (including one sequence derived from a tick sample) are most closely related to an L segment sequence derived from a historical 1969 clinical sample isolated in Tajikistan (KX013444). Two sequences, one from a clinical sample and one from a tick sample are most closely related to a 1990 clinical isolate sequence from Tajikistan (AY720893.2). Four partial Asia-2 L segment sequences (PV738567, PV738569, PV738571, PV738574) were excluded from phylogenetic analysis due to low reference coverage.

### The M segment displayed the lowest amino acid conservation within clinical and tick samples

**Fig 3:**
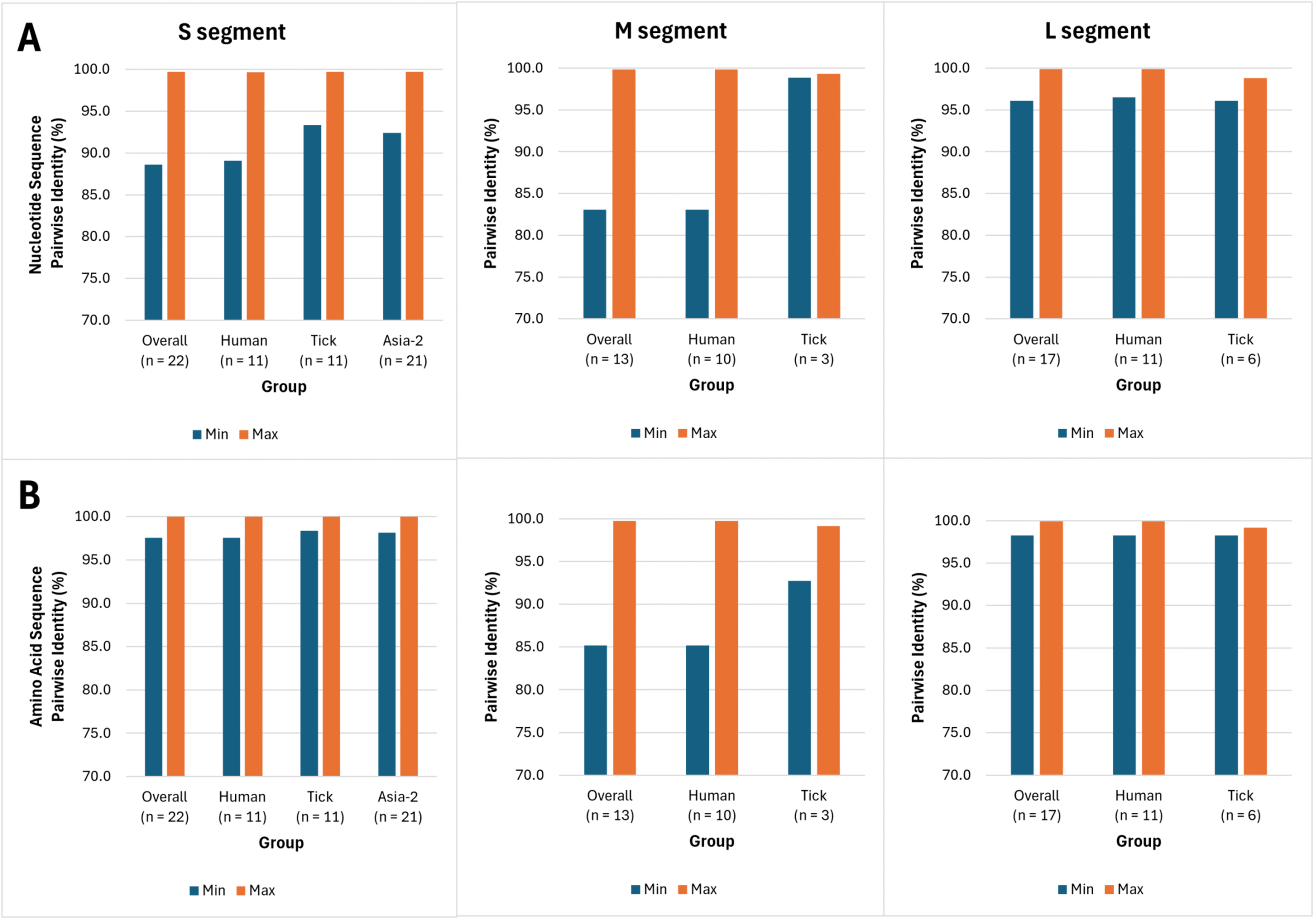
Within group minimum and maximum pairwise identity (%) for both nucleotide (A) and amino acid (B) sequences for each CCHFV genomic segment.

Minimum and maximum pairwise identities at both nucleotide and amino acid level are presented in Fig 3 for each sub-group. Since except for a single Asia-1 S segment sequence all other sequences were Asia-2, only the S segment Asia-2 within group pairwise identities are reported.

The average nucleotide sequence pairwise identity for the S segment was 94.9%, namely 94.7% for the human samples, 96.5% for the tick samples and 95.5% for the Asia-2 samples. For the M segment nucleotide sequences, the average pairwise identity was 91.2%, namely 90.7% for the human samples and 99.1% for the tick samples. For the L segment amino acid sequences, the average pairwise identity was 97.5%, namely 97.9% for the human samples and 97.4% for the tick samples.

The average amino acid sequence pairwise identity for the S segment was 99.3%, namely 99.4% for the human samples, 99.1% for the tick samples and 99.4% for the Asia-2 samples. For the M segment amino acid sequences, the average pairwise identity was 92.5%, namely 92% for the human samples and 96.1% for the tick samples. For the L segment amino acid sequences, the average nucleotide pairwise identity was 98.9%, namely 99% for the human samples and 98.8% for the tick samples.

## Discussion

The presence of CCHFV in Tajikistan has been known for decades, with the first records of clinical CCHF cases dating back to the 1940s, when CCHF was first recognised as a disease [25]. It was not until 2004 (for the S and M segments) and 2005 (for the L segment) that the first studies on genetic characterisation of circulating viruses in the region were published in the Russian literature [43–45], thus, providing evidence of the presence of Asia-2, and later in 2013 Asia-1 viruses, based on S segment analysis [27]. Only two historical strains isolated in 1969 [9] and 1990 [44–46] have been fully genetically characterised from the region, the former belonging to the Asia-2 genotype and the latter being an M segment Asia-1 reassortant virus.

Our study contributes significantly to the understanding of the circulation and genetic diversity of CCHFV in Tajikistan and more broadly in Central Asia by providing genomic insights into circulating CCHF viruses in clinics and ticks. Most of our genomic sequences cluster within the Asia-2 genotype for all genomic segments (Fig 2). A single S segment sequence from a clinical sample collected in Vahdat (C14) clustered within the Asia-1 genotype (Table 2, Fig 2A). The Asia-1 S segment had an average pairwise identity (%) of 89.66% and 97.96% at the nucleotide and amino acid level, respectively, with the other recovered Asia-2 S segment sequences. The incongruent phylogenetic pattern observed for the M and L segment sequences recovered from the same sample, which clustered with other published Asia-2 sequences, is consistent with S segment reassortment. The absence of other Asia-1 S segment sequences in our dataset together with the low proportion of minor variants sites (~ 0.5%) supports this as authentic viral reassortment rather than sample contamination or mixed infection. To our knowledge, this represents the first documented Asia-1 S segment reassortant virus identified in Tajikistan using full multi-segment genomic analysis, likely reflecting the historical scarcity of complete CCHFV genomic data from the region as well as the lower prevalence of Asia-1 viruses in Central Asia [47,48].

The functional implications of S-segment reassortment are potentially important. The S segment encodes the nucleoprotein (NP) which plays a central role in viral RNA encapsidation and replication, and is also the principal antigenic and diagnostic target for most serological and molecular detection assays [8]. Substitution of an Asia-1–derived S segment into an Asia-2 genomic background could therefore alter epitope composition and primer-binding efficiency, potentially influencing assay sensitivity or cross-reactivity. Although the Asia-1 and Asia-2 NP sequences share high amino-acid identity, the observed divergence at the nucleotide level (~10%) may affect the performance of lineage-specific RT-PCR or LAMP assays designed for Asia-2 viruses. These findings underscore the need for ongoing molecular surveillance to ensure that diagnostic reagents remain inclusive of circulating reassortant viruses.

Phylogenetic analysis of sequences from samples C19, C20, and C21 which were collected from patients who reported direct contact to C18 confirmed their genetic relatedness for all genomic segments (Fig 2). Notably, sequences from sample C16 which was collected from the same area three days earlier (Table 1) also cluster with these sequences (Fig 2), providing insights into a possible preceding transmission event between C16 and C18.

The lower average within group pairwise identity observed for the M segment at both nucleotide (Fig 3A) and amino acid level (Fig 3B), with averages of 91.2% and 92.5% respectively, is not surprising and is likely driven by immunological pressures [49]. Previous studies have also found the M segment to be the least conserved between different CCHFV genotypes at both nucleotide and amino acid levels [49], in accordance with our data. The relatively lower nucleotide pairwise identity of the S segment compared to the amino acid pairwise identity is indicative of a higher number of synonymous mutations in the S segment which translate into highly conserved nucleoproteins (NP).

One of the limitations of the study lies within our targeted amplicon-based enrichment design, which although highly effective for recovering near-complete CCHFV genomes from samples with low viraemia, was optimised for genotype IV (Asian lineages). As such, it may have limited sensitivity for more divergent genotypes, potentially biasing detection against strains from other genotypes. Due to the utilisation of a sensitive RT-PCR in our study, able to detect all CCHFV genotypes, the risks of false negatives have been mitigated. Nonetheless, to address the challenges associated with genetic divergence across CCHFV genotypes, we are developing a broader targeted sequencing panel incorporating conserved primer sets designed to capture all recognised CCHFV genotypes, enabling future studies to detect and characterise any emergent or translocated lineages in the region. Furthermore, while only *H. anatolicum* ticks have been collected in this study, further studies should aim to investigate other *Hyalomma* tick species such as *H. asiaticum* or genera such as *Rhipicephalus* ticks, which are present in the region and have been known to harbour different CCHFV genotypes [21,50].

This study provides critical insights into the genetic landscape of CCHFV in Tajikistan and highlights the value of integrating vector and human surveillance within a unified One Health framework to strengthen early detection of emerging CCHFV variants. By combining genomic data from both clinical and tick-derived samples, we demonstrate an approach that can reveal new transmission patterns, reassortment and movement of viral genotypes across ecological and administrative boundaries. Such integrated genomic monitoring provides an adaptable model for cross-border surveillance in Central Asia, where animal trade, pastoral mobility and shared vector habitats facilitate regional virus circulation. These efforts align directly with the WHO R&D Blueprint for Crimean-Congo haemorrhagic fever (2024–2030), which identifies integrated molecular surveillance, vector ecology, and data sharing across sectors as core priorities for global research coordination and outbreak preparedness. Strengthening this coordinated genomic surveillance capacity will be critical for anticipating viral evolution, refining diagnostics and guiding evidence-based control strategies at the human-animal-environment interface.

## Supporting information

S1 Table CCHFV enrichment scheme primer details (pool 1). (DOCX)

S2 Table CCHFV enrichment scheme primer details (pool 2). (DOCX)
