# Supplementary material for: Genomic characterisation of Crimean-Congo haemorrhagic fever virus (CCHFV) in Tajikistan identifies a novel reassortant virus
Source: PLoS Negl Trop Dis. 2026 Apr 7;20(4):e0014204. doi: 10.1371/journal.pntd.0014204 (PMC13075792; doi:10.1371/journal.pntd.0014204)
Supplement: S1 Table — (DOCX) [file pntd.0014204.s001.docx]

Table S1A - CCHFV enrichment scheme primer details (pool 1).

| **Primer Name** | **Sequence (5’-3’)** | **Final Concentration in pool (µM)** | **Pool ID** |
| --- | --- | --- | --- |
| CCHFV_A_S1_L | TCTCAAAGAAACACGTGCCGC | 6 | S1 |
| CCHFV_A1_S1_R | AGGACTTTGTGGCTTAAGGCTG | 6 | S1 |
| CCHFS_1_RIGHT_B | AGGACTTTGTTACTCAAAGCTG | 2 | S1 |
| CCHFV_A1_S3_L | CATAAGGACGAGGTTGACAAAGC | 2 | S1 |
| CCHFV_A1_S3_R | ACAATTGTCTTGGCACACGGAT | 2 | S1 |
| CCHFV_A_M1_L | ACTTTGAGGAAGTGGATTGAGCA | 2 | M1A |
| CCHFM_1_LEFT_B | GCTTTGAGAGAGTGACTTAGGCA | 2 | M1A |
| CCHFV_A_M1_R | TGACTGAAAGYAGACTCCTCAC | 2 | M1A |
| CCHFM_1_RIGHT_B | AGACCGACAGTAAACCCCTTGC | 2 | M1A |
| CCHFV_A_M3_L | TGGAAATAATCTTAACKTTAYCTCAGGG | 2 | M1A |
| CCHFM_3_LEFT_B | TGGAAATAATCTTGACGTTACCTCAAGG | 2 | M1A |
| CCHFV_A_M3_R | GAGAAGCACATTGATTTCAACCTCT | 2 | M1A |
| CCHFM_3_RIGHT_B | GAGAAGCACATTGATTTCAATCTCT | 2 | M1A |
| CCHFV_A_M5_L | TCTGGTGATGACTGCATATCAA | 2 | M1A |
| CCHFV_A_M5_L_B | TCTGGTGACGACTGTATATCGA | 2 | M1A |
| CCHFV_A_M5_L_B2 | AGGTGACGACTGCATATCGAGG | 2 | M1A |
| CCHFV_A_M5_R | CCATGGTTGTCTTGGGGTGATT | 2 | M1A |
| CCHFM_5_RIGHT_B | CCATAGTTGTTTTAGGATGATT | 2 | M1A |
| CCHFV_A_M5_R_B2 | AGTTGTTTTGGGATGATTTGAC | 2 | M1A |
| CCHFV_A_M7_L | TGAAYTTAGAGAGAATTCCCTGGG | 2 | M1A |
| CCHFM_7_LEFT_B | TGAACTTGGAAAGAATTCCTTGGA | 2 | M1A |
| CCHFV_A_M7_R | GTTGCYCTGAAACACATGCG | 2 | M1A |
| CCHFV_A_M9_L | ACAAGCTTRTCRATTGAGGCACC | 2 | M1A |
| CCHFV_A_M9_R | TCTGTCTTAATGTACTCAACTTTCCACT | 2 | M1A |
| CCHFV_A_M11_L | AACGGACACCTRATTCATAAAATTGA | 2 | M1A |
| CCHFM_11_LEFT_B | AATGGGCATATGATTCACAAAATTGA | 2 | M1A |
| CCHFV_A_M11_R | TCAAGCTTCCTYGCCATGAGAC | 2 | M1A |
| CCHFV_A_M13_L | TCTTTATGTTTGGATGGAGGATCC | 2 | M1A |
| CCHFV_A_M13_R | GCACGCAGTCAAGRTTTAGGTC | 2 | M1A |
| CCHFV_A_L1_L | TTACCCACGTTGACACAGACAG | 2 | L1 |
| CCHFV_A_L1_R | AGAGCGTCAAAATGCGTTTGTC | 2 | L1 |
| CCHFL_1_RIGHT_B | AGAGCATCAAAGTGTGTCTGTC | 2 | L1 |
| CCHFV_A_L3_L | ACTGCATGTTCAACTGCAAACT | 2 | L1 |
| CCHFV_A_L3_R | AGCTTGTTTATCTGCTTCCCGA | 2 | L1 |
| CCHFL_3_RIGHT_B | AGCTTGTTTATCTGTTTCCCAA | 2 | L1 |
| CCHFV_A_L5_L | TCTTTAAGAAGAAGGACATCATGAAATTCA | 2 | L1 |
| CCHFL_5_LEFT_B | TCTTTAAGAAGAAGGACATTATGAAGTTCA | 2 | L1 |
| CCHFV_A_L5_R | GATCTCTGAACTCTGGATGCAGT | 2 | L1 |
| CCHFL_5_RIGHT_B | AATCTCTGAACTCTGGATGTAGT | 2 | L1 |
| CCHFL_5_RIGHT_C | AATCTCTGAACTCTGGATGTAAT | 2 | L1 |
| CCHFV_A_L7_L | CYTTCAAGGACTATGGAGAAAGAGG | 2 | L1 |
| CCHFL_7_LEFT_B | CTTTCAAGGACTATGGAGAGAGGGG | 2 | L1 |
| CCHFV_A_L7_R | ARTCCTTTACGCATTGCCTGTT | 2 | L1 |
| CCHFV_A_L9_L | TGGCGTRAACATTAGCAACGTA | 2 | L1 |
| CCHFL_9_LEFT_B | CGGTGTGAACATTAGCAACATA | 2 | L1 |
| CCHFV_A_L9_R | GGGCACCTTTAGGTCAAACAATAT | 2 | L1 |
| CCHFL_9_RIGHT_B | AGGCACCTTTAAATCAAACAATAT | 2 | L1 |
| CCHFV_A_L11_L | ACGGGAAAATATGTGARACCTTCC | 2 | L1 |
| CCHFL_11_LEFT_B | ATGGTAAAATATGCGAAACCTTCT | 2 | L1 |
| CCHFV_A_L11_R | GCTTATAAAGTGGTCTCTGCTGC | 2 | L1 |
| CCHFV_A_L13_L | CCTTTGTGAACAGCGACAGACA | 2 | L1 |
| CCHFV_A_L13_R | GTGTGGCTAGGATTCTTYCGTA | 2 | L1 |
| CCHFL_13_RIGHT_B | GTGTGACTGGGATTTTTCCGTA | 2 | L1 |
| CCHFV_A_L15_L | CAGCACGGTAGATGGTCTAACA | 2 | L1 |
| CCHFL_15_LEFT_B | TAGCACTGTGGATGGTTTAACA | 2 | L1 |
| CCHFV_A_L15_R | GCTTTGACAATTCCAGGTGCTG | 2 | L1 |
| CCHFL_15_RIGHT_B | GCTTTGATAATTCCAGATGTTG | 2 | L1 |
| CCHFV_A_L17_L | TTATTCACATCCAATGGCGAGC | 2 | L1 |
| CCHFL_17_LEFT_B | CTATTTACATCCAATGGTGAAC | 2 | L1 |
| CCHFV_A_L17_R | GGCCGTCATCTGATGTTGTCTT | 2 | L1 |
| CCHFL_17_RIGHT_B | GGCCATCATCAGGTGTAGTTTT | 2 | L1 |
| CCHFV_A_L19_L | CAGGCAGCATTAAGAAGATCCT | 2 | L1 |
| CCHFL_19_LEFT_B | CAGGCAGTATTAAAAAGATCTT | 2 | L1 |
| CCHFV_A_L19_R | GCCCATCATGAACTCGCTGTAA | 2 | L1 |
| CCHFL_19_RIGHT_B | GCCCATCATAAATTCACTATAA | 2 | L1 |
| CCHFV_A_L21_L | CTCKGGGTTAGTTAGCTCAGCG | 2 | L1 |
| CCHFL_21_LEFT_B | CTCTGGATTAGTCAGCTCAGCA | 2 | L1 |
| CCHFV_A_L21_R | TGTCRTCTCTATTCAATGATGCCT | 2 | L1 |
| CCHFV_A_L23_L | AGCTCTCGCTTCCTATATATACGATATT | 2 | L1 |
| CCHFV_A_L23_R | AGTCMACCTTCGTAGGCTTGAC | 2 | L1 |
| CCHFL_23_RIGHT_B | AATCAACCTTGGTGGGTTTAAC | 2 | L1 |
| CCHFV_A_L25_L | GGAAGGCAGGTTTTATAGGCTCT | 2 | L1 |
| CCHFL_25_LEFT_B | GGAAGGTAGGTTCTATAGGCTTT | 2 | L1 |
| CCHFV_A_L25_R | GCTCATCCAAGAAAGCACTTGC | 2 | L1 |
| CCHFV_A_L27_L | TGATGAGACWGACACTCAGACCA | 2 | L1 |
| CCHFV_A_L27_R | CTCACATTGGTGTCTAACGTAA | 2 | L1 |
| CCHFV_A_L29_L | GGGAAGAATATCCTTGTTCGTGCA | 2 | L1 |
| CCHFV_A_L29_R | AGCCAAGATTGCCTGTTCTGAG | 2 | L1 |
| CCHF_TAJ_M1_L | AACTTTGAGTGAGTGACTTGAGCA | 4 | M1B |
| CCHF_TAJ_M1_R | ACTGACAGTAGACCCCTTGCA | 4 | M1B |
| CCHF_TAJ_M3_L | AAGACACTGAAGGCCTGCTAGA | 2 | M1B |
| CCHF_TAJ_M3_R | CACAGACATGCGATTTGATCAGC | 2 | M1B |
| CCHF_TAJ_M5_L | GCAAGGCATCTTCTGGTTCCA | 2 | M1B |
| CCHF_TAJ_M5_R | AATAGTGGGATGTTGGGGCATC | 2 | M1B |
| CCHF_TAJ_M7_L | TCTTGCCAGACATGTGATGCA | 6 | M1B |
| CCHF_TAJ_M7_R | TTTTCTTGCAGATGCTCAAGTGC | 6 | M1B |
| CCHF_TAJ_M9_L | TGTGCTCAGGAAACCCTTGTTTT | 2 | M1B |
| CCHF_TAJ_M9_R | ACAGCAGGTACATCCAGTGC | 2 | M1B |
| CCHF_TAJ_M11_L | CCTACAGGTCTACCACATAGGAAAC | 2 | M1B |
| CCHF_TAJ_M11_R | GTCTGGGTCTTCACTCTTCACATG | 2 | M1B |
| CCHF_TAJ_M13_L | CCACAGAGCATTTTAATCGAGCAC | 2 | M1B |
| CCHF_TAJ_M13_R | ATGACAGTGTGATATGGGTGCG | 2 | M1B |
