# Supplementary material for: Genomic characterisation of Crimean-Congo haemorrhagic fever virus (CCHFV) in Tajikistan identifies a novel reassortant virus
Source: PLoS Negl Trop Dis. 2026 Apr 7;20(4):e0014204. doi: 10.1371/journal.pntd.0014204 (PMC13075792; doi:10.1371/journal.pntd.0014204)
Supplement: S2 Table — (DOCX) [file pntd.0014204.s002.docx]

Table S1B - CCHFV enrichment scheme primer details (pool 2).

| **Primer Name** | **Sequence (5’-3’)** | **Final Concentration in pool (µM)** | **Pool ID** |
| --- | --- | --- | --- |
| CCHFV_A1_S2_L | ATTACCAACAGGCTGCTCTCAA | 2 | S2 |
| CCHFV_A_S2_R | GTTTTTGTAAAGCTCCTGTGCC | 2 | S2 |
| CCHFV_A1_S4_L | TCCTTCCAGCAGAACAGGATCT | 2 | S2 |
| CCHFS_4_LEFT_B | TCTTTCCAGCAGAACAGGATCT | 2 | S2 |
| CCHFV_A1_S4_R | TCAAAGATATCGTTGCCGCACA | 2 | S2 |
| CCHFV_A_M2_L | GAGACACCTCCACACCAACAGT | 6 | M2A |
| CCHFM_2_LEFT_B | AGGACACTCCCACATCGACAAC | 6 | M2A |
| CCHFV_A_M2_L_C | GGGATACTCTCACATCGACAGC | 2 | M2A |
| CCHFV_A_M2_R | TCAGTGTCYTCCTCTAAGGTGA | 2 | M2A |
| CCHFM_2_RIGHT_B | TCAGTGTCTTCTTCCAAGGTAA | 2 | M2A |
| CCHFV_A_M4_L | CTAGRATAGCTGACACRCCTGGG | 2 | M2A |
| CCHFV_A_M4_L_B | CTAGAATAGCGGATACACCTGGA | 2 | M2A |
| CCHFV_A_M4_R | TTRTCACCYGGACCACCATA | 2 | M2A |
| CCHFV_A_M6_L | GTTCAGGTGGGCAGCAACATT | 2 | M2A |
| CCHFV_A_M6_R | GCCAGCTGCTYCTTTTTAATRC | 2 | M2A |
| CCHFM_6_RIGHT_B | GCCAACTGCTCCTCTTTAGTGC | 2 | M2A |
| CCHFV_A_M8_L | CATCAGCTGCACTTGAGCATCT | 2 | M2A |
| CCHFV_A_M8_R | GTTCMACTGARCTCCAACTAAGTGC | 2 | M2A |
| CCHFV_A_M10_L | GTAATCCYACTTGGTGCTGGGG | 2 | M2A |
| CCHFV_A_M10_R | CCAGTCTCCCATGTTRCAGTAG | 2 | M2A |
| CCHFM_10_RIGHT_B | CCAATCCCCCATATTACAGTAG | 2 | M2A |
| CCHFV_A_M12_L | GATGAACCAGATGAACTTACAGT | 2 | M2A |
| CCHFV_A_M12_R | AGGCCTCTGGTTCTTCTGCA | 2 | M2A |
| CCHFV_A_L2_L | GTGATGAAGTGGAAGCAGGCAT | 2 | L2 |
| CCHFV_A_L2_R | CCGCAACGACATAACTTCACCA | 2 | L2 |
| CCHFV_A_L4_L | TGAACTGCTGTATAAGGCACCT | 2 | L2 |
| CCHFL_4_LEFT_B | TGAACTGTTGTATAAGGCACCC | 2 | L2 |
| CCHFV_A_L4_R | AGGCTAGACTTRCTTAAATAAAGGT | 2 | L2 |
| CCHFL_4_RIGHT_B | AGGCTGGACTTGCTCAAATAAAGGT | 2 | L2 |
| CCHFV_A_L6_L | GACATCAAACCTAGYCTGACCA | 2 | L2 |
| CCHFV_A_L6_R | AGCTGCCTTGTTTCCAGTTGAT | 2 | L2 |
| CCHFV_A_L8_L | CTGCAACAAAAACCCCTGTGTC | 2 | L2 |
| CCHFV_A_L8_R | TTGCTATCCTCAAAGCCTTTGT | 2 | L2 |
| CCHFV_A_L10_L | AGGATTATTCAACTTATGCAAGAGGAGTA | 2 | L2 |
| CCHFV_A_L10_R | TGTCACAGTGCCTTACCTTGAC | 2 | L2 |
| CCHFV_A_L12_L | CTTCTGCTCTTGAAGGTAGATTCG | 2 | L2 |
| CCHFL_12_LEFT_B | CTTCTGCACTTGAGGGTAGATTTG | 2 | L2 |
| CCHFV_A_L12_R | CCAGAGCATATGCCTTTCTGCT | 2 | L2 |
| CCHFV_A_L14_L | CCATTCAATGATTATCAGCAAGCA | 2 | L2 |
| CCHFL_14_LEFT_B | CCCTTTAATGACTATCAGCAGGCA | 2 | L2 |
| CCHFV_A_L14_R | CAATGTTTTTCTTRACTAATTCTTTCCA | 2 | L2 |
| CCHFL_14_RIGHT_B | CTATGTTTTTTTTGACTAATTCCTTCCA | 2 | L2 |
| CCHFV_A_L16_L | AGGTCCAGAAATTCATTTATCACCTT | 2 | L2 |
| CCHFL_16_LEFT_B | AGGTTCAGAAATTCATTTATCACCTG | 2 | L2 |
| CCHFV_A_L16_R | GCAACTTTTCATCAGTAGTCTTGTGT | 2 | L2 |
| CCHFL_16_RIGHT_B | GCAACTTCTCGTCAGTGGTTTTGTGC | 2 | L2 |
| CCHFV_A_L18_L | GCACCTAAAGCACAGCTAGGAG | 2 | L2 |
| CCHFV_A_L18_R | TGACTGTGTCATTTCCATCCCA | 2 | L2 |
| CCHFV_A_L20_L | TGAAACGTTTTGGAAGCATGCC | 2 | L2 |
| CCHFV_A_L20_R | GCTACTWGACTGGATTGATACACTRT | 2 | L2 |
| CCHFV_A_L22_L | GGCTGAGAGATGGTAAGAGAAGAG | 2 | L2 |
| CCHFV_A_L22_R | CTACCAGTCAATTGTTTGCCGC | 2 | L2 |
| CCHFV_A_L24_L | TCAGCAACTGAACAGGTTCGAC | 2 | L2 |
| CCHFV_A_L24_R | GCAGCGAAGACTTATCTGCTTG | 2 | L2 |
| CCHFL_24_RIGHT_B | GCAATGAGGACTTGTCTGCTTG | 2 | L2 |
| CCHFV_A_L26_L | CATTGCAGGAAAGTTRCACATT | 2 | L2 |
| CCHFL_26_LEFT_B | CATTGCAGGGAAACTGCACATC | 2 | L2 |
| CCHFV_A_L26_R | TCCTTGAGAGGACGACTTTGGA | 2 | L2 |
| CCHFV_A_L28_L | GGCTTCAACAATGTGCTTGTCA | 2 | L2 |
| CCHFV_A_L28_R | TGACTACTCTCTCAGGCTTTGACT | 2 | L2 |
| CCHFV_A_L30_L | ACAAGGCTAGATTACAATAAGTTGATTGA | 2 | L2 |
| CCHFL_30_LEFT_B | ACAAGGCTAGACTACAACAAATTAATTGA | 2 | L2 |
| CCHFV_A_L30_R | YCCCCACACCCTAGAAATAATCA | 2 | L2 |
| CCHF_TAJ_M2_L | GTCAGTCACCAGCACAAAGC | 2 | M2B |
| CCHF_TAJ_M2_R | CTTCATTGAAGTGACCCTCCCC | 2 | M2B |
| CCHF_TAJ_M4_L | GCGTCAGTCTTTAAAGAGCATAGAGA | 2 | M2B |
| CCHF_TAJ_M4_R | AGTTCACTGCCCAATCTCTGATC | 2 | M2B |
| CCHF_TAJ_M6_L | TGATACCAAAGGGCACTGGAGA | 2 | M2B |
| CCHF_TAJ_M6_R | TTTCCTAACAACCCAGGGGATTC | 2 | M2B |
| CCHF_TAJ_M8_L | TGAATCTCACAAATGTTACTGTAGCCT | 2 | M2B |
| CCHF_TAJ_M8_R | TCCCCATGGTGCCTCAATTGA | 2 | M2B |
| CCHF_TAJ_M10_L | GCACATCATCAACCTGCCTACA | 2 | M2B |
| CCHF_TAJ_M10_R | CCAGGAAGTGTTGAAGTGTGGT | 2 | M2B |
| CCHF_TAJ_M12_L | GATATCAGGTTTGAAGTTTGCAAGCT | 2 | M2B |
| CCHF_TAJ_M12_R | GGAGCAAGGAACAAGAAAATCCC | 2 | M2B |
